# Interprofessional education in primary care for the elderly: a pilot study

**DOI:** 10.1186/1472-6920-13-161

**Published:** 2013-12-05

**Authors:** Barth Oeseburg, Rudi Hilberts, Truus A Luten, Antoinette VM van Etten, Joris PJ Slaets, Petrie F Roodbol

**Affiliations:** 1 Wenckebach Institute, School of Nursing and Health, University Medical Centre Groningen, University of Groningen, P.O. Box 30.001, 9700 RB Groningen, The Netherlands; 2 Wenckebach Institute Postgraduate School of Medicine, University Medical Centre Groningen, University of Groningen, P.O. Box 30.001, 9700 RB Groningen, The Netherlands; 3 Primary Care Practice Stationsstraat Hoogeveen, Stationsstraat 6a, 7901 AB Hoogeveen, The Netherlands; 4 University Medical Centre Groningen, University of Groningen, P.O. Box 30.001, 9700 RB Groningen, The Netherlands; 5 Geriatric Medicine, University Medical Centre Groningen, University of Groningen, P.O. Box 30.001, 9700 RB Groningen, The Netherlands

**Keywords:** Interprofessional, Learning, Primary care, Physician, Nursing

## Abstract

**Background:**

The Dutch health care system faces huge challenges with regard to the demand on elderly care and the competencies of nurses and physicians required to meet this demand.

At present, the main focus of health care in the Netherlands lies on illness and treatment. However, (frail) elderly need care and support that takes their daily functioning and well-being into consideration as well. Therefore, health care professionals, especially those professionals working in primary care such as GPs and practice nurses, will be challenged to a paradigm shift in emphasis from treating illness to promoting health (healthy ageing). Interprofessional education is necessary to realise this shift in professional behaviour. Evidence indicates that interprofessional education (IPE) can play a pivotal role in enhancing the competencies of professionals in order to provide elderly care that is both effectively, integrated and well-coordinated. At present, however, IPE in primary care is rarely utilised in the Netherlands. Therefore, the aim of this pilot study was to develop an IPE program for GPs and practice nurses and to evaluate the feasibility of an IPE program for professionals with different educational backgrounds and its effect on the division of professionals’ tasks and responsibilities.

**Methods:**

Ten GPs and 10 practice nurses from eight primary care practices in two provinces in the north of the Netherlands, Groningen and Drenthe (total population about 1.1 million people), participated in the pilot IPE program. A mixed methods design including quantitative and qualitative methods was used to evaluate the IPE program.

**Results:**

During the program, tasks and responsibilities, in particular those related to the care plan, shifted from GP to practice nurse. The participants’ attitude toward elderly (care) changed and the triage instrument, the practical tool for prioritising preferences of the elderly and discussing their medication use, was considered to have an added value to the development of the care plan.

**Conclusions:**

The results of this pilot study show that an interprofessional education program for professionals with different educational backgrounds (GPs and practice nurses) is feasible and has an added value to the redefining of tasks and responsibilities among GPs and practice nurses.

## Background

The Dutch health care system faces huge challenges with regard to the demand on elderly care and the competencies of nurses and physicians required to meet this demand, especially in primary care. However, the various parties involved (the elderly, professionals, policy makers) feel that the competencies they currently possess are insufficient to meet the increasingly complex needs of the elderly [1-6]. The number of elderly persons (> 65 years) in the Netherlands (total population of about 16.7 million people) is growing rapidly from about 2.5 million to 4.1 million in 2030. In addition, the number of frail elderly is likely to increase between 2010 and 2030 from about 650,000 to over one million [2]. Approximately 95% of the elderly live independently at home and are registered with a general practitioner (GP). In turn, approximately 25% of the elderly who live independently are frail [2].

As a consequence of the growing number of elderly, the need for complex care will also increase.

At present, health care in the Netherlands focuses mainly on illness and treatment. In addition, (frail) elderly have expressed unmet needs regarding daily functioning and well-being. Therefore, health care professionals, especially in primary care, will be challenged to a paradigm shift in emphasis from treating illness to promoting health (healthy ageing) [2-6].

To meet the needs of the (frail) elderly and to optimise their daily functioning and well-being, while at the same time controlling the increasing costs, a well-structured and fully integrated care system is needed. Care should be organised in the desired living environment of the elderly, which, in most cases, will be their own homes. The system needs to focus on the following aspects [7-9]: prevention of physical, psychological, and social problems on an individual and group level; early detection and comprehensive assessment of physical and psychosocial needs; the delivery of effective care arrangements covering a wide range of health care and community services; coordination of care and interprofessional cooperation; ongoing follow-up of the elderly; productive interaction between the elderly and professionals to empower the elderly to manage and adapt to ageing; and promoting healthy ageing and well-being.

Ideally, primary care professionals, such as GPs and practice nurses (registered nurses or practice assistants with vocational education employed by GPs), should play a central role in the care for the elderly [2,5,6,10]. GPs already play a key role in the Dutch health care system and function as gatekeepers for other community and institutional services. A substantial number of GPs employ practice nurses in their practices, particularly for the care given to chronically ill patients, e.g. patients with diabetes or asthma/COPD. Care to these groups is based on cooperation and coordination between GP and practice nurse and involves shared responsibilities and adequate specifications of responsibilities delegated from GP to practice nurses. However, as mentioned before, the provision of care to these groups is mainly focused on treating illness and does not meet the needs of the (frail) multimorbid elderly [2,3,6-9]. The organisation of the care for complex patients needs to be defragmented in order to meet the new demands [2,4-9].

To realise a well-structured and fully integrated primary care system, a shift in professional behaviour, particularly in the domains of proactive/preventive care, coordination of care, and communication and cooperation with the elderly and other professionals, is necessary. In addition, a redesign of tasks and responsibilities of GPs and practice nurses is expected to improve the quality of elderly care [2,5-9]. Professional behaviour is inextricably linked to the education of professionals. However, the curricula for initial and secondary education for professionals are not suited to educate professionals in the competencies that are necessary for elderly care, because these curricula focus mainly on disease-related competencies and competencies relevant to their own profession [11-13]. Changing professional behaviour and initiating a fully integrated and well-coordinated provision of elderly care, with shared responsibilities and adequate specifications of delegated responsibilities, requires interprofessional education (IPE) [2,5,6,14]. Evidence indicates that IPE can enhance the competencies of professionals, which will lead to an improvement in the quality of health care and better patient outcomes [15,16]. At present, however, IPE in primary care is rarely utilised in the Netherlands. Therefore, a pilot study was initiated. The aim of this pilot study is to develop an IPE-program for GPs and practice nurses and to evaluate both the feasibility of an IPE program for professionals with different educational levels and the effect such a program will have on the division of their tasks and responsibilities.

## Methods

### Intervention

An IPE program, based on a social constructivist approach and consisting of four half-day shared sessions, was developed [17]. The social constructivist approach emphasises the collaborative nature of learning. Learning is an active process, embedded in social and physical contexts in which learners construct their own competencies based on prior competencies. Cooperation with others creates the opportunity to define or refine learners’ understanding and to create shared understandings with respect to the division of tasks and responsibilities between GPs and practice nurses.

During the IPE program, GPs and practice nurses prepared themselves for the shared education sessions by reading relevant literature and the GP and practice nurse prepared practical assignments based on cases generated from their own local practice. Experts gave short lectures and led the plenary sessions in which the practical assignments were discussed and reflected on.

Draft versions of the IPE program were discussed with expert group (GPs, practice nurses, geriatrician). The educational aim of the program was to realise a shift in tasks and responsibilities from GP to practice nurse.

The following objectives were outlined for the sessions:

Session 1: Vision on elderly care and triage. The aim of this session was: to examine knowledge of and attitudes toward the elderly and elderly care; to explore the use of a comprehensive Web-based triage screening instrument, based on the INTERMED [18-20], the ‘Groningen Frailty Indicator’ [21,22], and the Groningen Well-being Indicator [23]; and to collect data on the medical, psychosocial, and functional capabilities and limitations of all elderly patients in the participating primary care practices.

Session 2: Care plan. The aim of the second session was to develop a comprehensive care plan based on the care plan developed by the Dutch College of General Practitioners [24] and a practical tool to prioritise preferences of the elderly and discuss their medication use, based on Fried et al. [25].

Session 3: Thinking in groups. In this session, elderly patients were empirically categorised into five meaningful segments (primary segmentation) with different health-related needs: vital problems, psychosocial coping problems, physical and mobility problems, problems in multiple domains, and problems caused by extremely frailty. These segments are characterised by the significant relations found with gender, age, frailty, bio-psychosocial complexity, living arrangements, well-being, and preferred decisional control [26]. Segmenting the elderly based on their needs offers GP and practice nurse the possibility to intervene proactively; not only on an individual level but also on a group level. A proactive intervention plan can prevent health problems in the elderly and can help keep chronically ill patients as vital as possible.

Session 4: Reflection and feedback on the IPE program. In this session the final practical assignment (session 3) was discussed and reflected on. In addition, the IPE program was evaluated with the participants and appointments were made for further evaluation.

### Participants and procedure

A convenience sample of 10 GPs and 10 practice nurses from eight primary care practices in two provinces in the north of the Netherlands, Groningen and Drenthe, (total population about 1.1 million people) participated. Six primary care practices were informed of the project during a meeting on a transition experiment in elderly care in Groningen in which they participated. Two primary care practices (in Drenthe) were informed by one of the project members and received additional educational materials.

A mixed methods design including quantitative and qualitative methods was used to evaluate the IPE program. The division of tasks and responsibilities of GPs and practice nurses was measured by a VAS scale. The following indicators were measured: case finding, the assessment of medical and psychosocial functioning and recording, medication, the development of a comprehensive care plan, discussion with the elderly on the care plan, execution of the care plan, consultation of other professionals in health and community care, and monitoring the care (plan).

The score on each indicator could range from 0 (tasks and responsibilities of the practice nurse) to 10 (tasks and responsibilities of the GP). For example, a score of score 5 indicated full cooperation between GP and practice nurse. Primary care practices (the GP and practice nurse) were asked to rate the division of tasks and responsibilities before and during the program and to state their future preferences. Four of the eight primary care practices responded.

The quality of the program was measured by a questionnaire developed by the Wenckebach Institute aimed at evaluating educational programs. This questionnaire is based on Kirkpatrick’s model of evaluating training programs [27] and measures the quality of the following indicators: added value of the lectures; clarity, practicability, and added value of the practical assignments; and suitability of the program to facilitate change within practices. The score on each indicator can range from 0 (strongly disagree) to 5 (strongly agree).

In addition to filling in the questionnaire, the participants were asked to report positive features of the program and to give advice on how to improve the program. The response rate was 60% (N = 20). Finally, semi-structured telephone interviews were conducted with primary care practices (GPs and practice nurses) which addressed the following issues: the participants’ expectations with regard to the program; changes in their attitude with regard to elderly and elderly care; suitability of the program to facilitate change within practices; change, or intentions to change tasks and responsibilities of the GP and practice nurse; and advice to improve the program. All the interviews with both GPs and practice nurses were tape-recorded and transcribed. Six out of eight primary care practices responded (response rate 75%). In total, six GP’s and six practice nurses were interviewed.

### Analysis

The raw descriptive data of the VAS scale were used to analyse the division of tasks and responsibilities of the primary care practices (N = 4) before and during the program and to list their wishes regarding the division of tasks and responsibilities in the future. Next, the mean score and standard deviation were calculated for the scores obtained on the Wenckebach Institute’s quality questionnaire. Subsequently, scores for each session [1-3] were calculated. Finally, the recorded telephone interviews were transcribed for analysis. Two researchers independently analysed and categorised the data into the themes that structured the interview [28].

### Ethical approval

The project was funded by a grant from ZonMW (The National Care for the Elderly Program: 310300003; The Netherlands Organisation for Health Research and Development) as well as by the University Medical Centre Groningen (UMCG). The study was presented to the ethical review board of the UMCG, which did not find further approval necessary.

## Results

### Tasks and responsibilities

During the IPE program, a shift in tasks and responsibilities from GPs to practice nurses in the primary care practices took place, especially with regard to tasks and responsibilities related to the care plan. A shift in tasks and responsibilities between GP and practice nurses on case finding and medication did not occur during the IPE program. In addition, in most primary care practices there is a need for a greater shift in tasks and responsibilities on activities with regard to the care plan from GP to practice nurses in the future (Table 1).

**Table 1:** Tasks and responsibilities GPs and practice nurses before, during the program and desirable in the future (N = 4 primary care practices)

	**0**^**1 **^**PN**^**2**^	**1**	**2**	**3**	**4**	**5 Both**	**6**	**7**	**8**	**9**	**10 GP**
*Case finding*											
Before					1	3					
During					1	3					
Future						4					
*Assessment of: medical and psychosocial functioning and recording*											
Before					1	2	1				
During					1	3					
Future					2	2					
*Medication*											
Before						1	1	2			
During						1	1	2			
Future						1	1	2			
*Development of a comprehensive care plan*											
Before				1		1		1			
During				2		1					
Future	1			1		1					
*Execution of the care plan*											
Before					1	1		1			
During				1	1	1					
Future		1	1			1					
*Consultation of other professionals in health and community care*											
Before				2		1	1				
During				2	1	1					
Future			2	1		1					
*Monitoring the care(plan)*											
Before					1	3					
During				1		3					
Future			2	1		1					

1. Score range: 0 (tasks and responsibilities of the practice nurse) to 10 (tasks and responsibilities of the GP); the score 5 indicated (full cooperation between GP and practice nurse).

2. PN = practice nurse.

### Quality of the program

The mean scores on the Wenckebach Institute’s quality questionnaire on the three indicators in session one and two all ranged from 3 (neither agree nor disagree) to 4 (agree). In session three, the mean scores ranged from 3 to 2 (disagree) (Table 2). However, most deviations of the means are considerably large, indicating considerable variation in the answers of the respondents.

**Table 2:** Means and standard deviations on the Wenckebach Institute quality of the program questionnaire (N = 12)

	**Session 1**		**Session 2**		**Session 3**	
	**Vision elderly care/triage**		**Care plan**		**Thinking in groups**	
	***Mean***	***sd***^***1***^	***Mean***	***sd***	***Mean***	***sd***
Added value of the lectures	3.94^2^	.33	3.67	.39	3.00	.71
Clarity, practicability and added value of the practical assignments	3.32	.75	3.60	.63	2.64	.84
Suitability of the program to facilitate change within practices	3.82	.87	3.41	.63	2.82	.98

1. sd = standard deviation.

2. score range: 0 (strongly disagree) to 5 (strongly agree), score 3 indicates not disagree/not agree.

### Expectation

Despite their willingness to participate in the IPE program, five of the interviewed participants (N = 12) indicated that they did not have any explicit expectations of the IPE program. The other seven participants expressed expectations with respect to learning more about using the triage instrument and learning to interpret and manage the data on the functioning of the elderly in their own primary care practices. Participants also expected to be offered practical tools and evidence-based interventions for handling problems specific to the elderly.

### Changes in attitude

Most of the interviewed participants indicated that the IPE program changed their attitudes toward the elderly and care for the elderly. Key insights gained from the program included the importance of taking the patient’s perspective into account in the planning of care; being more proactive and preventing problems instead of being reactive and solving problems; becoming more attentive to the needs of the elderly due to the risk of multimorbidity; and realising that elderly care comprises more than just disease management. Four participants also indicated that the collaboration with other disciplines and other primary care practice led to a change in their attitudes toward elderly care.

### Suitability of the program and change within practices

Most of the interviewed participants indicated that the lectures and practical assignments with regard to the triage instrument and the care plan had already initiated a shift in tasks and responsibilities from GP to practice nurse or that there was at least an incentive to realise this shift. However, most participants also pointed out that they required more concrete information and training on the following items of the program: the triage instrument, individual care plans, practical tools and evidenced based interventions for handling certain problems in the elderly, and the availability of health care and community resources that can be linked up to primary care.

All of the interviewed participants indicated that the lecture and practical assignment on thinking in groups (segments of elderly) was too scientific and not directly suitable for primary care practice. Another issue that became apparent during the sessions was that the participants did not have a procedure in mind for dealing with complex patients, nor was the number of such patients in their own practices known to them.

One participant indicated that the IPE program had no added value at all.

### Advice to improve the program

The participants offered several suggestions for improving the program. Twelve participants desired more information and training on: the interpretation and management of the data generated by the triage instrument; the development of comprehensive care plans, practical tools, and evidenced- based interventions for handling problems specific to the elderly; training in communication skills; lectures and training on moral dilemmas; and information on the availability of health care and community resources to link up to primary care. They also expressed a need for practical tools for the prevention and management of (potential) health problems in the elderly (individual level and segmentation level).

## Conclusion and discussion

The results of this pilot study show that an interprofessional education (IPE) program for professionals with different educational levels, in particular GPs and practice nurses in primary care, is feasible and has an added value to the redefining of tasks and responsibilities.

During the program, tasks and responsibilities, in particular with respect to the care plan, shifted from GP to practice nurse. The program had a positive impact on the participants’ attitude toward elderly (care), and the triage instrument in particular was considered to have an added value to the development of the care plan.

Despite the fact that the IPE program was developed in close cooperation with expert groups, the program did not entirely meet the expectations of the participants. The length of the program, four half day sessions, was deemed too short to adequately increase the knowledge on, for example, the interpretation of the data generated by the triage instrument. The program was also too short to address the needs of the participants regarding practical tools and evidenced based interventions to handle certain problems in the elderly. Furthermore, participants found the information on the IPE program too concise, and GPs did not inform their practice nurses sufficiently about the program’s content. Indeed, this latter point could have influenced the expectations of the participants and the subsequent success of the program [29].

However, this was a pilot study, and one characteristic of a pilot study is that participants are both subjects and developers of the intervention at the same time. The results of this pilot study and the participants’ suggestions for improvement will be used to develop an adapted interprofessional education program for GPs and practice nurses.

### Strengths and limitations

The strength of this study is that it is the first study in the Netherlands that focuses on interprofessional education in primary care practice. Another strength was that the program was developed in collaboration with the target groups.

However, this pilot study was limited in both scale and scope. Therefore, the findings should be interpreted with caution. First, the participants were recruited via our network and could be characterised as innovators [30]. Second, we did not employ a comparison group and the number of participants was also limited. Third, the VAS scale used to measure a shift in tasks and responsibilities from GP to practice nurse was developed specifically for the program and has not been validated in research as yet. Finally, factors influencing the shift of tasks and responsibilities were not evaluated, and the effects on the health care system and patient outcomes were not included in the pilot study.

### Findings in relation to other studies

To our knowledge, there is a paucity of literature on interprofessional education specifically pertaining to GPs and practice nurses in primary elderly care [31]. Our results are in line with the limited research on interprofessional learning in primary care and reviews on interprofessional education [14,31,32].

A study by Pearson & Pandya [31], for example, found that primary care professionals value interprofessional education and the sharing of knowledge and expertise. In keeping with a recent review of Reeves et al. [14] on the effectiveness of interprofessional education, our pilot study shows a change in the attitudes of the participants and their performance in practice: level 1 – 2/3 Kirkpatrick’s model [27]. As mentioned above, the impact of the IPE program on the health care system itself and on patient outcomes was not measured in our pilot and could therefore not be compared with findings in other studies.

The participants in this pilot study mentioned some barriers to the success of the IPE program. In the literature, other barriers are also mentioned that hinder the implementation of an interprofessional education program. These barriers include the social identity of professional groups, hierarchical relations between professionals, lack of time, workload, and lack of financial incentives for the education program and for interprofessional collaboration in practice. In addition, factors related to the implementation and change process of professionals and practices such as the support of senior management, dynamic leadership, inclusion of all staff members, a proactive approach to prevent resistance, and sustaining change during and after the initial implementation process are important [10,30,33-37].

However, despite these start-up problems, collaboration between professionals is crucial in today’s increasingly complex healthcare system. Although the literature indicates that the link between interprofessional education and interprofessional collaboration is not clear, working on clarifying this link is worthwhile [10,38-41]. There is a need for theory-driven development and implementation of interprofessional education programs, combined with high quality research on the effects of interprofessional education. Future research is necessary to learn more about the effects of interprofessional education on an individual level, i.e. how professionals learn in certain settings and why some are more capable than others; as well as its effects on an organisational level, i.e. how factors such as the organisation of care, financial incentives, costs, and patients outcomes influence the health care system [16,30,33,39-41].

## Competing interest

The authors declare that they have no competing interest.

## Authors’ contributions

All six authors have approved the final manuscript, accept full responsibility for the design and the conduct of the study, and approve the decision to publish. All six authors have had full access to all the data in the study and take responsibility for the integrity of the data and the accuracy of the data analysis. All six authors have made contributions to the study design, acquisition, and interpretation of the data. BO drafted the manuscript and RH, TAL, AVMvE, JPJS, and PFR have made substantial contributions to writing the manuscript. All authors read and approved the final manuscript.

## Pre-publication history

The pre-publication history for this paper can be accessed here:

http://www.biomedcentral.com/1472-6920/13/161/prepub
